# Unveiling miRNA30b's Role in Suppressing ADAM12 to Combat Triple-Negative Breast Cancer

**DOI:** 10.1155/2024/5202941

**Published:** 2024-10-30

**Authors:** Qing-hua Yin, Jian-bing Hu, Qiang Zhou, Jie Weng, Er-dong Shen, Fang Wen, Song-lian Liu, Lei-lan Yin, Ya-jun Tong, Ling Long, Ke-wei Tang, Si-te Bai, Lu-di Ou

**Affiliations:** Department of Oncology, Yueyang Central Hospital, Yueyang 414000, Hunan, China

**Keywords:** ADAM12, invasion, migration, miRNA30b, proliferation, triple-negative breast cancer

## Abstract

**Background:** Triple-negative breast cancer, a subtype of breast cancer, is characterized by a poor prognosis. Recent studies have shown that miRNA30b acts as an oncogene and is vital for the proliferation of malignancies across various systems. This study aimed to elucidate the impact of miRNA30b on the proliferation, migration, and invasion capabilities of breast cancer cells *in vitro*.

**Methods:** Triple-negative breast cancer cell lines MDA-MB-231 were transiently transfected with miRNA30b inhibitor, mimic, or the negative control by Lipofectamine 2000. Successful transfection was confirmed by quantitative real-time polymerase chain reaction (qRT-PCR). Functional assays, including CCK8, clone formation, scratch, and transwell assays, were conducted to evaluate the proliferation, invasion, and migration ability of MDA-MB-231 cells in each group. The target protein of miRNA30b was determined using an online prediction data website, and the dual-luciferase assay confirmed whether there was a binding site between miRNA30b and ADAM12. The effect was further verified by Western blot analysis.

**Results:** MDA-MB-231 cells were transfected with miRNA30b inhibitor, mimic, and negative control. miRNA30b expression was downregulated in the cells. Relative to the negative control group, miRNA30b expression significantly increased in the mimic group and decreased in the miRNA30b inhibitor group, with the differences being statistically significant. The miRNA30b mimic group exhibited a significant increase in miRNA30b expression and a corresponding promotion of cell proliferation, colony formation, and migration. Conversely, the miRNA30b inhibitor group displayed significantly reduced miRNA30b expression and suppressed cell proliferation, colony formation, and migration abilities compared to the negative control cells. Bioinformatics software predicted ADAM12 as a potential target of miRNA30b. Dual-luciferase assays confirmed the presence of a binding site between miRNA30b and ADAM12. Western blot analysis revealed that overexpression of miRNA30b downregulated ADAM12 expression in MDA-MB-231 cells.

**Conclusions:** miRNA30b could promote proliferation, migration, and invasion of TNBC cell lines MDA-MB-231. The effect of miRNA30b on triple-negative breast cancer would be achieved partly at least through inhibiting the expression of ADAM12.

## 1. Introduction

Women's lives are impacted by the very prevalent and persistent disease known as breast cancer. One of the subtypes of breast cancer that lacks the progesterone receptor gene, the estrogen receptor gene, and the human epidermal growth factor receptor 2 gene is known as triple-negative breast cancer. Triple-negative breast cancer has a worse median and overall survival rate than other subtypes of breast cancer due to its high likelihood of recurrence, treatment resistance, and metastasis [1]. Finding possible therapeutic targets to prevent tumor spreading and developing new triple-negative breast cancer treatment regimens are therefore crucial.

Numerous studies have found that microRNAs play an important role in invasion, treatment resistance, and the development of cancer [2, 3]. Endogenous single-stranded RNA molecules with a length of around 22 nucleotides are referred to as miRNAs [4]. miRNAs have been linked to biological processes such as tumor cell growth, differentiation, metabolism, and death, according to earlier research [5]. The expression of miRNA30 was markedly downregulated in breast tumor–initiating cells. In addition to preventing breast tumor beginning cells from self-renewing, overexpression of miRNA30 can also prevent the expression of the target gene ITGB3, which causes apoptosis in breast tumor–initiating cells [6]. The miRNA30 family can also control the expression of genes linked to apoptosis and proliferation, which can impact the growth of breast cancer cells [7]. The miRNA30 family includes miRNA30b, whose expression is intimately linked to the development of several malignant tumors, including breast, colon, prostate, and cervical cancers [8, 9]. The role of miRNA30b in breast cancer has received less attention. It demonstrates a tissue type–dependent way, meaning that miRNA30b could have diverse functions in various malignancies. TNBC's probable roles and mechanisms, however, remain unidentified.

A disintegrin and metalloproteinase 12 (ADAM12) is closely associated with tumor development or prognostic parameters [10–12], is highly expressed in a variety of cancers (lung [13, 14], breast [15–18], bladder [19], liver [20], etc.), and has potential as a tumor diagnostic and prognostic marker [21, 22], according to an increasing number of studies. ADAM12 performs a wide range of biological functions, including cell–cell and cell–matrix interactions as well as involvement in a number of illnesses. In addition, it contributes to the emergence of several disorders. It has been demonstrated that human breast cancer cells are one of the malignant tumors that significantly damage women's health [23]. When exposed to hypoxia, ADAM12 expression is elevated, which stimulates EGFR signaling and speeds up the migration and invasion of breast cancer cells [24, 25].

In this study, the triple-negative breast cancer MDA-MB-231 cell line was transfected with miRNA30b inhibitors, mimics, and negative controls. We confirmed that miRNA30b modulated cell proliferation, invasion, and migration by targeting ADAM12 in TNBC cells. Our results indicate that miRNA30b may play a role as a novel oncogene in TNBC.

## 2. Methods

### 2.1. Materials and Reagents

Breast cancer cell line MBA-MD-231 was purchased from Shanghai Saibaikang Biotechnology; 10% fetal bovine serum was purchased from Hangzhou Tianhang Biotechnology Co., Ltd; DMEM high glucose medium was purchased from HyClone, Inc; Lipofectamine 2000 was purchased from Invitrogen, Inc.; and miRNA30b inhibitor, mimic, and its negative control were purchased from Guangzhou Ruibo Biotechnology Co. Ltd. ADAM12/β-actin/miRNA30b primers were synthesized by Wuhan Jinkairui Bioengineering Co., Ltd; reverse transcription polymerase chain reaction (PCR) kit was purchased from Wuhan Kelu Biotechnology Co., Ltd; real-time quantitative PCR kit was purchased from Wuhan Kelu Biotechnology Co., Ltd; and plasmid pGL6-miRNA/pRL-TK and dual-luciferase reporter gene detection kit was purchased from Shanghai Biyuntian Biotechnology Co. Rabbit anti-human ADAM12 was purchased from Wuhan Sanying Biotechnology Co. CCK8 reagent was purchased from Biosharp Biotechnology (lot number: BS350b), crystal violet powder was purchased from Shanghai Huitian Laboratory Equipment Company Limited (lot number: 71012314), and transwell chamber was purchased from Corning Incorporated (lot number: CLS3422). The real-time fluorescence PCR instrument was purchased from Life Technologies, the multifunctional enzyme labeling instrument was purchased from Tecan (Switzerland), the fluorescence inverted microscope was purchased from Olympus (Japan), and the CO_2_ incubator was purchased from SHEL LAB (USA).

### 2.2. MDA-MB-231 Cell Culture

Human triple-negative breast cancer cell line MBA-MD-231 cells were digested using DMEM containing 10% fetal bovine serum in a constant temperature and humidity sterile cell culture incubator at 37°C, with 5% CO_2_ as well as humidity maintained at 95%. MBA-MD-231 cells were walled cells, and the cells were treated with trypsin digestion solution in the biosafety cabinet and passaged once every 1 day.

### 2.3. Cellular Transfection

One day before transfection, 5 × 10^5^ cells per well (6-well plate) were inoculated in 2 mL of medium. 250 μL of the Opti-MEM serum-free medium was used to dilute the miRNAs to 200 nM, and then gently mixed, and incubated for 5 min at room temperature. Before use, Lipofectamine 2000 was shaken gently, 5 μL of the Lipofectamine 2000 was taken and diluted with 250 μL of Opti-MEM serum-free medium, and incubated for 5 min at room temperature. The miRNA diluted in the first 2 steps and Lipofectamine 2000 were (total volume 500 μL) gently mixed and left at room temperature for 20 min. 500 μL of the transfection solution and 1500 μL of the basal medium were added to each well of cells and shaked gently. The cells were incubated at 37°C in a CO_2_ incubator, and after 6 h of transfection, the cells were exchanged for a complete medium.

### 2.4. Real-Time qPCR

After the samples were processed according to the instructions, total RNA was extracted by TRIpure Total RNA Extraction Reagent. First-strand RNA was reverse transcribed using the M-MLV Reverse Transcriptase (ELK Biotechnology, EQ002) kit. Fluorescence quantitative PCR was performed using the SYBR Premix Ex Taq kit according to the instructions. Relative expression was analyzed by the 2^−ΔΔct^ method. miRNA30b's internal reference was H-U6, and Hsa-miRNA30b's primer sequences were as follows: 5′-CTCAACTGGTGTCGTGGAGTCGGCAATTCAGTTGAGAGAGCTGAGT -3′ (RT primer); 5′-GGCCCTGTAAACATCCTACAC-3′ (upstream primer); and 5′-GGCCCTGTAAACATCCTACAC-3′ (downstream primer). H-U6 primer sequences were as follows: 5′-AACGCTTCACGAATTTGCGT-3′ (RT primer); 5′-CTCGCTTCGGCAGCACAT-3′ (upstream primer); and 5′-AACGCTTCACGAATTTGCGT-3′ (downstream primer).

### 2.5. CCK8 Assay

The cell suspension of MBA-MD-231 at the logarithmic growth stage was inoculated with 1 × 10^5^ cells/mL per well in a 96-well culture plate. Five replicate wells were setup for each group and placed in the cell culture incubator for 24 h. The culture medium was changed and 10 μl of the CCK8 reagent solution was added to each well and the culture was continued for 2.0 h. The absorbance value was measured at 450 nm.

### 2.6. Plate Clone Formation Assay

After transfection, MDA-MB-231 cells were taken from the 6-well plate and reinoculated into the 6-well plate at 500 cells/well. 2 mL of DMEM complete medium was added to each well, 3 replicate wells were setup in each group, and placed in 37°C, 5% CO_2_ incubator for 2 weeks. The medium was discarded, washed twice with PBS, and fixed with anhydrous ethanol for 15 min. After air-drying, the plates were stained with 0.1% crystal violet for 20 min, washed with tap water, air-dried, and photographed. The clone formation rate is derived as follows: clone formation rate = amount of clones/number of inoculated cells × 100%.

### 2.7. Scratch Assay

MDA-MB-231 cells were transfected in 6-well plates and incubated for 24 h. The cells were incubated for 24 h. The surface of the cell culture was marked vertically with a 200 μL gun, washed twice with PBS, and replaced with DMEM complete medium containing 2% FBS, 2 mL was added to each well, and three replicate wells were set up for each group. The cell migration rate was observed between the groups.

### 2.8. Transwell Assay

The breast cancer cell line MBA-MD-231 from each group after transfection was treated with trypsin digest, made into a cell suspension, and the cell concentration was adjusted to 1 × 10^5^ cells/mL and 3 replicate wells were set up for each group. Cells were inoculated into the upper chamber of the transwell lined with Matrigel gel, and 600 μL of DMEM with 10% fetal bovine serum was placed in the lower chamber and incubated in the incubator for 24 h. The culture fluid in the upper chamber was aspirated, excess Matrigel gel and nonmigrated cells were carefully wiped off with a cotton swab, anhydrous methanol was added to fix the cells for 25 min, and then the crystalline violet solution was stained for 25 min and observed under a microscope.

### 2.9. Prediction of Target Genes

The potential target genes of miRNA30b were predicted by bioinformatics technology websites' (TargetScan, miRwalk, and miRanda) database. The corresponding target genes were selected for validation.

### 2.10. Dual-Luciferase Carrier Assay

MDA-MB-231 cells were spread in 24-well plates at 0.5–2 × 10^5^ cells/well, and when the cells reached 30%–50% confluence, they were replaced with double antibody–free serum-free DMEM high sugar medium at 250 μL per well. pGL6-miRNA-ADAM12-3′UTR-WT + pRL-TK was incubated with Lipofectamine 2000 according to the instructions. pGL6-miRNA-ADAM12-3′UTR-WT + pRL-TK, mimics NC + pGL6-miRNA-ADAM12-3′UTR-WT + pRL-TK, miRNA mimics + pGL6-miNAR-ADAM12-3′UTR-WT + pRL-TK, pGL6-miRNA-ADAM12-3′UTR-Mut + pRL-TK, mimics NC + pGL6-miRNA-ADAM12-3′UTR-Mut + pRL-TK, and miRNA30b mimics + pGL6-miRNA- ADAM12-3′UTR-Mut-3′UTR + pRL-TK cells were transfected separately. After transfection for 24 h, the medium was discarded. Precooled PBS was washed 2 times and proteins were collected with protein lysate. A 24-well plate was taken, protein at 30 μL/well and firefly luciferase substrate at 100 μL/well as an internal reference were added, mixed well, and the value of firefly luciferase activity was detected. Then, the termination solution and sea kidney luciferase substrate were added, and the sea kidney luciferase activity value was assayed. The formula to calculate luciferase activity is as follows: luciferase activity of the reporter plasmid per well = sea kidney luciferase activity value/firefly luciferase activity value. Each set of experiments was repeated 3 times.

### 2.11. Western Blotting Analysis

After transfection, MDA-MB-231 cells were taken from 6-well plates and the medium was discarded. The cells were washed twice with prechilled PBS, and 80 μL of RIPA cell lysate was added to each well to extract the total protein. The bicinchoninic acid (BCA) kit was used to determine the protein concentration and quantify the protein samples to be measured. The quantified protein samples were subjected to electrophoresis and membrane transfer. After transferring, the samples were closed with 5% skimmed milk for 1 h. The strips were incubated with primary antibody diluted at 1:1000 and placed in a refrigerator at 4°C overnight. Three washes of PBST were performed for 10 min each time, and the secondary antibody was diluted at 1:1000 for 45 min and 3 washes of PBST for 10 min each time. β-Actin was used as the internal reference protein. We expose the film, followed by development and fixation, then wash it with water, dry, and finally scan to analyze the results.

### 2.12. Statistical Processing

In this study, SPSS 20.0 statistical software was utilized for analysis. Data were expressed as means ± standard, and each experiment was repeated 3 times. Real-time quantitative PCR data were calculated by the 2^−ΔΔct^ method, and comparisons between two groups were analyzed using the student *t*-test, and one-way ANOVA was performed to compare three or more groups. GraphPad Prism 9.0 was used for charting and statistical significance was determined at *p* < 0.05.

## 3. Results

### 3.1. Expression of miRNA30b

**Figure 1:**
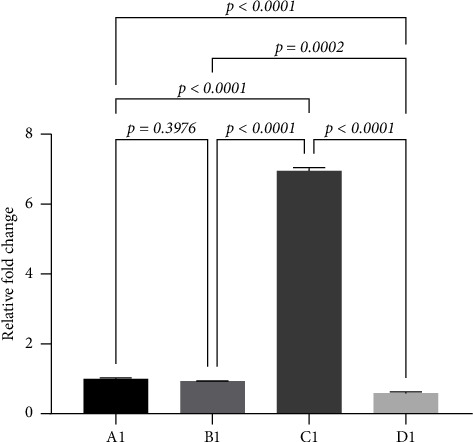
Relative expression levels of miRNA30b in transfected MDA-MB-231 cells. A1: MBA-MD-231, B1: MBA-MD-231 + miRNA30b mimic NC, C1: MBA-MD-231 + miRNA30b mimic, and D1: MBA-MD-231 + miRNA30b inhibitor. Data are represented as mean ± SD. The test was conducted using the one-way ANOVA test and the *p* values are presented in numerical form in the graph.

We employed real-time fluorescence quantification to assess the expression levels of miRNA30b in MDA-MB-231 breast cancer cells transfected with miRNA30b mimic NC, miRNA30b mimic, and miRNA30b inhibitor. The results indicated that the expression of miRNA30b was significantly reduced in the cells transfected with the miRNA30b inhibitor (0.59 ± 0.04, *p* < 0.0001). Conversely, miRNA30b expression was significantly elevated in the miRNA30b mimic-transfected group (6.95 ± 0.09, *p* < 0.0001) compared to the negative control group (1.00 ± 0.02) (Figure 1).

### 3.2. The Effect of miRNA30b on the Proliferation Ability of Breast Cancer Cells

**Figure 2:**
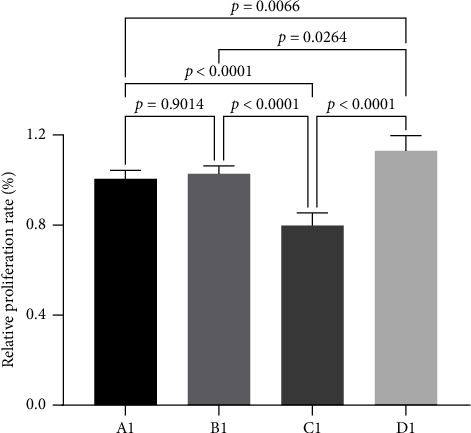
CCK8 detection of cell proliferative activity. A1: MBA-MD-231, B1: MBA-MD-231 + miRNA30b mimic NC, C1: MBA-MD-231 + miRNA30b mimic, and D1: MBA-MD-231 + miRNA30b inhibitor. Data are represented as mean ± SD. The test was conducted using the one-way ANOVA test and the *p* values are presented in numerical form in the graph.

The breast cancer cell line MBA-MD-231 was transfected with miRNA30b mimic, miRNA30b inhibitor, and negative control miRNA30b mimic NC, followed by a CCK8 assay after 24 h of culture. The results indicated a significant reduction in the cell number of MBA-MD-231 transfected with miRNA30b mimic compared to the miRNA30b mimic NC (79.72% ± 5.58% vs. 100.27% ± 3.35%, *p* < 0.0001). Conversely, the cell number of MBA-MD-231 transfected with miRNA30b inhibitor significantly increased (112.90% ± 6.74% vs. 100.27% ± 3.35%, *p*=0.0066). These findings suggest that miRNA30b markedly decreases the proliferation of MBA-MD-231 cells and plays a significant role in the growth regulation of breast cancer cells (Figure 2).

### 3.3. The Effect of miRNA30b on the Clonogenic Ability of Breast Cancer Cells

**Figure 3:**
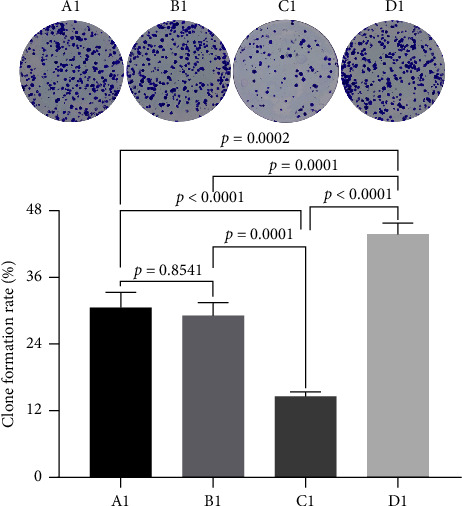
Plate cloning assay to detect the clone-forming ability of cells. A1: MBA-MD-231, B1: MBA-MD-231 + miRNA30b mimic NC, C1: MBA-MD-231 + miRNA30b mimic, and D1: MBA-MD-231 + miRNA30b inhibitor. Data are represented as mean ± SD. The test was conducted using the one-way ANOVA test and the *p* values are presented in numerical form in the graph.

We utilized the plate clone formation assay to further assess the impact of miRNA30b on the proliferative capabilities of MBA-MD-231 breast cancer cells. The findings indicated that the number of clones in the MBA-MD-231 cells transfected with the miRNA30b mimic in the experimental group was significantly lower than that in the control group transfected with the miRNA30b mimic NC (14.53% ± 0.90% vs. 29.27% ± 2.23%, *p*=0.0001). Conversely, the number of clones in the cells treated with the miRNA30b inhibitor was significantly higher compared to those in the control group transfected with a negative control (34.87% ± 1.92% vs. 29.27% ± 2.23%, *p*=0.0001) (Figure 3). Therefore, miRNA30b appears to diminish clonal formation efficiency in these cells.

### 3.4. The Effect of miRNA30b on the Lateral Migration Ability of Breast Cancer Cells

**Figure 4:**
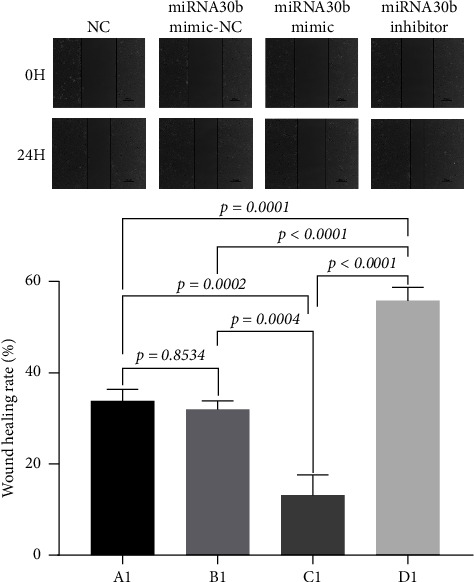
Scratch assay to detect the migratory ability of cells. A1: MBA-MD-231, B1: MBA-MD-231 + miRNA30b mimic NC, C1: MBA-MD-231 + miRNA30b mimic, and D1: MBA-MD-231 + miRNA30b inhibitor. Data are represented as mean ± SD. The test was conducted using the one-way ANOVA test and the *p* values are presented in numerical form in the graph.

We investigated the effects of miRNA30b on the migration capabilities of breast cancer MBA-MD-231 cells. In the scratch assay, the observed changes in scratch width result from both cell migration and proliferation. The results revealed that 24 h postscratch, the healing rate in the experimental group transfected with the miRNA30b mimic was significantly slower compared to the negative control group (13.41% ± 4.24% vs. 31.89% ± 2.02%, *p*=0.0004). Conversely, the healing rate in the experimental group transfected with the miRNA30b inhibitor was significantly faster compared to the negative control (55.78% ± 3.08% vs. 31.89% ± 2.02%, *p* < 0.0001) (Figure 4). These findings indicate that miRNA30b inhibits the migration of breast cancer cells.

### 3.5. The Effect of miRNA30b on the Invasive Ability of Breast Cancer Cells

**Figure 5:**
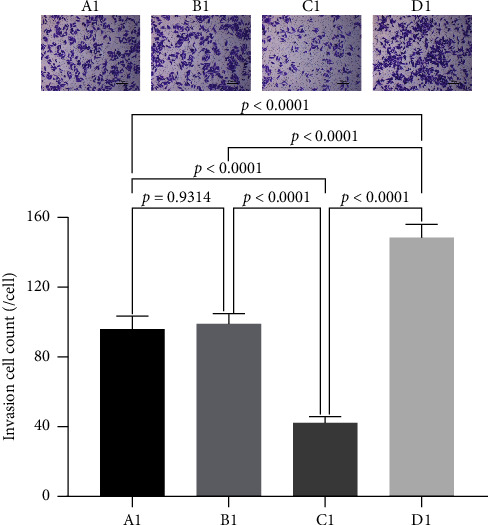
The transwell invasion assay detects the invasive ability of cells. A1: MBA-MD-231, B1: MBA-MD-231 + miRNA30b mimic NC, C1: MBA-MD-231 + miRNA30b mimic, and D1: MBA-MD-231 + miRNA30b inhibitor. Data are represented as mean ± SD. The test was conducted using the one-way ANOVA test and the *p* values are presented in numerical form in the graph.

We further assessed the impact of miRNA30b on the longitudinal migration of breast cancer cells using a transwell assay. The results demonstrated that the number of MBA-MD-231 breast cancer cells transfected with the miRNA30b mimic that penetrated the membrane was significantly lower compared to the NC group transfected with the same mimic (42.06% ± 3.51% vs. 98.82% ± 6.15%, *p* < 0.0001). Conversely, the penetration of cells transfected with the miRNA30b inhibitor in the experimental group was significantly higher than that in the negative control group (148.50% ± 7.46% vs. 98.82% ± 6.15%, *p* < 0.0001) (Figure 5). This suggests that miRNA30b plays a significant role in inhibiting the migration ability of breast cancer cells.

### 3.6. Predicted Results of miRNA30b Target Genes

**Figure 6:**
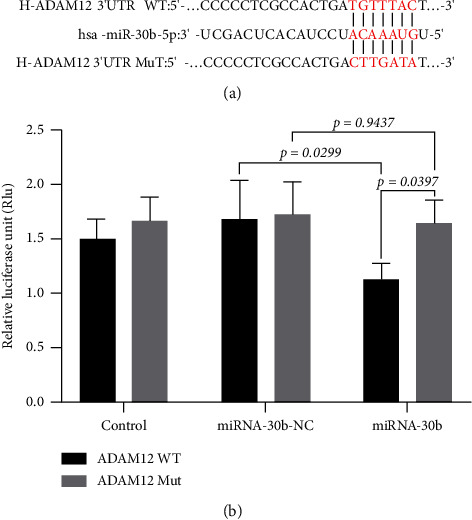
Dual-luciferase vector assay confirms targeted binding of miRNA30b to the ADAM12 gene. (a) miRNA30b and ADAM12 binding sites predicted by bioinformatics. (b) miRNA30b mimic/miRNA negative control and ADAM12-3′-UTR wild-type/ADAM12-3′-UTR mutant vectors were constructed and co-\transfected for relative fluorescein activity (sea kidney fluorescein/firefly fluorescein). UTR mutant vectors were cotransfected and assayed for relative fluorescein activity (sea kidney fluorescein/firefly fluorescein). Data are represented as mean ± SD. Comparisons between the two groups were made using the *t*-test, and comparisons between multiple groups were made using the one-way ANOVA test, and the *p* values are presented in numerical form in the graph.

miRNA30b was predicted to regulate ADAM12 gene expression based on bioinformatics technology sites (TargetScan, miRWalk, and miRanda) as shown in Figure 6(a).

### 3.7. Dual-Luciferase Vector Assay Confirms Targeted Binding of miRNA30b to ADAM12 Gene

The dual-luciferase vector assay revealed that the luciferase activity in MDA-MB-231 cells transfected with miRNA30b mimics + pGL6-miRNA-ADAM12-3′UTR-WT + pRL-TK was significantly higher compared to cells transfected with the negative control sequence mimics NC + pGL6-miRNA-ADAM12-3′UTR-WT + pRL-TK (1.14 ± 0.13 vs. 1.68 ± 0.35, *p*=0.0299). However, the luciferase activity in cells transfected with miRNA30b mimics + pGL6-miRNA-ADAM12-3′UTR-Mut + pRL-TK was not significantly different from that in cells transfected with mimics NC + pGL6-miRNA-ADAM12-3′UTR-Mut + pRL-TK (1.64 ± 0.21 vs. 1.72 ± 0.30, *p*=0.9347) (Figure 6(b)). This confirms that miRNA30b can directly bind to ADAM12-3′UTR, indicating that ADAM12 is a direct target of miRNA30b.

### 3.8. Effect of miRNA30b on ADAM12 Protein Expression in MDA-MB-231 Cells

**Figure 7:**
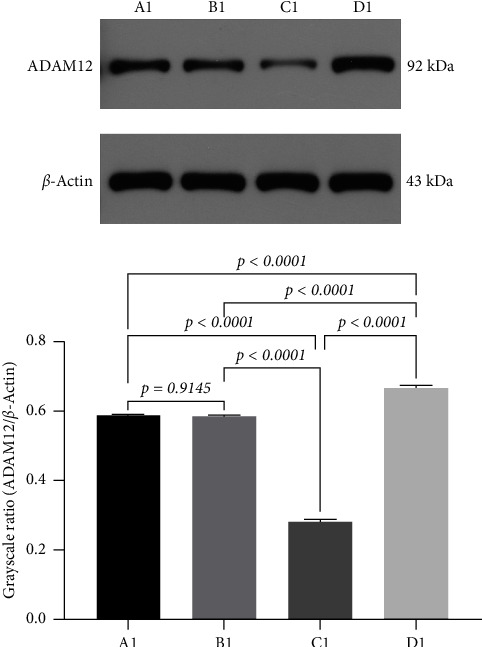
Western blotting for ADAM12 protein expression in MDA-MB-231 cells after transfection with miRNA30b mimic, miRNA30b-NC and miRNA30b inhibitor. A1: MBA-MD-231, B1: MBA-MD-231 + miRNA30b mimic NC, C1: MBA-MD-231 + miRNA30b mimic, and D1: MBA-MD-231 + miRNA30b inhibitor. Data are represented as mean ± SD. The test was conducted using the one-way ANOVA test and the *p* values are presented in numerical form in the graph.

To further confirm the direct targeted link between miRNA30b and ADAM12. We investigated the protein-level expression of the target gene ADAM12 modulated by miRNA30b in breast cancer cells. The protein expression of ADAM12 in MDA-MB-231 cells transfected with miRNA30b mimic was significantly lower compared to the negative control group transfected with miRNA30b mimic NC (0.28 ± 0.01 vs. 0.58 ± 0.01, *p* < 0.0001). Conversely, the protein expression of ADAM12 was significantly elevated in the group treated with the miRNA30b inhibitor relative to the miRNA30b mimic NC group (0.67 ± 0.01 vs. 0.58 ± 0.01, *p* < 0.0001, Figure 7). In other words, miRNA30b facilitated the malignant progression of breast cancer by downregulating the expression of ADAM12.

## 4. Discussion

Breast cancer is the number one malignant tumor threatening women's health worldwide, seriously endangering women's life and health, and triple-negative breast cancer is a type of breast cancer with the highest mortality rate and a hotspot of current research. The aim of this paper is to investigate the effect of miRNA30b on the proliferation and invasion of breast cancer cells and the possible mechanisms. The experimental results of this study showed that in breast cancer cells MDA-MB-231 cells, miRNA30b may inhibit cell proliferation and migration by downregulating the expression of ADAM12, and then inhibit cell proliferation and migration. With the deepening research, miRNA30b has the potential to become a molecular target for breast cancer treatment.

Since the discovery of the first miRNA-lin4 in nematodes by Victor Ambros in 1993 [26], and the term microRNA was coined in 2001 [27], the study of miRNAs has made great progress, and more and more biological functions of miRNAs have been discovered, which has laid a good foundation for the clinical application of miRNAs. A large number of literature studies have shown that it mediates posttranscriptional regulation of genes and has a close relationship with the occurrence and development of human diseases, especially in tumors. miRNAs are abnormally expressed in tumor cells, which has an important impact on the proliferation, apoptosis, migration, invasion, and other processes of tumor cells. miRNAs are closely associated with the occurrence and development of breast cancer. A retrospective study on lymph node–negative breast cancer showed that four subtypes of breast cancer could be identified by clustering analysis of miRNA expression profiles, and triple-negative breast cancer was the most prominent because of its unique miRNA profile [28]. Cascione et al. [29] published a large-scale study of triple-negative breast cancer, which found that between primary triple-negative breast cancer and normal breast tissue, a total of 116 miRNA expression imbalance occurred, and a total of 6 miRNAs were differentially expressed between triple-negative breast cancer lymph node metastasis and normal tissues. Thus, it can be seen that there is a correlation between miRNAs and triple-negative breast cancer, and the high and low changes in their expression may be the basis for diagnosis, treatment, or prognosis of triple-negative breast cancer. The miRNA30 family, a significant group within the miRNA complex, encompasses miRNA30a, miRNA30b, miRNA30c, miRNA30d, and miRNA30e. These members share a high degree of sequence homology and are likely to exert a crucial regulatory influence on the development and progression of breast cancer [5, 8].

MiRNA30b, whose coding gene is localized at 8q24.22, has attracted increasing attention in terms of its relevance to breast cancer and its biological function. Silencing of CDH11 or ITGA5 in breast cancer patients with ER/PR negative breast cancer cells recapitulated inhibitory effects of miRNA30 on skeletal tumor burden [30]. A valuable therapeutic tool for the monitoring of individuals in need of early and more effective therapy, MiRNA30b may be able to identify breast cancer patients who are more likely to have disease progression. When compared to samples from healthy breasts, the expression of miRNA30b in breast cancer patient tissues was considerably lower [31]. As opposed to healthy donors, breast cancer patients had considerably greater levels of the circulating miRNA30b [32]. In addition, patients with new metastases and patients with positive axillary lymph nodes had considerably higher levels of circulating miRNA30b [32]. Roy et al. [33] showed that the chemotherapeutic agent trastuzumab can upregulate the expression of miRNA30b in breast cancer treatment and can achieve clinical therapeutic effects by targeting the cell cycle protein E2 (cyclin E2 [CCNE2]), leading to the blockage of the G1 phase of the cell, inhibition of cell proliferation, and increase of cell apoptosis. Wang et al. [34] found that miRNA30b and ITGβ3 were abnormally expressed in breast cancer tissues. miRNA30b overexpression significantly downregulated the expression of ITGβ3, which inhibited the proliferation, invasion, and apoptosis of breast cancer cells. This is consistent with our findings.

According to Wang et al. [34], induced overexpression of miRNA30b increased MDA-MB-231 and HCC1937 cell proliferation, migration, and invasion while decreasing cell apoptosis. Furthermore, miRNA30b could directly target ASPP2 to activate the AKT signaling pathway. Overexpressing miRNA30b significantly decreased the capacity of SKBR3 and MDA-MB-231 cells to proliferate, move, and invade, according to the research by Abraham et al. [35]. In SKBR3 and MDA-MB-231 cells, miRNA30b overexpression is expected to regulate apoptosis-related proteins (Bcl-2, Bax, active caspase-3, and caspase-9), and this is thought to occur through suppressing the PI3K/Akt signaling pathway by targeting Derlin-1. Their research further supports our results.

Bioinformatics tools predicted that the target gene of miRNA30b is ADAM12, which has been reported to be overexpressed in many tumors [36, 37], especially in breast cancer, and is thought to play an important role in carcinogenesis [38, 39]. Studies have demonstrated that ADAM12 is overexpressed in triple-negative breast cancer tissues and cell lines and that the overexpression of ADAM12 in triple-negative breast cancer is partially mediated by DNA hypomethylation, an epigenetic alteration that has a significant impact on OS in triple-negative breast cancer patients [40]. Previous studies have confirmed that ADAM12-L 3′UTR is a direct target of miRNA29 and miRNA200 family members. ADAM12 levels were significantly upregulated in the claudin-low cell line, while the level of ADAM12 in this cell line was not statistically different from its levels in other cell lines. The selective upregulation of ADAM12 in claudin-low cell lines may be due to the fact that ADAM12 is inhibited by one or more miRNAs, and its common target may be the 3′UTR of ADAM12, which is the miRNA29 family^41^. In this study, miRNA30b was found to significantly reduce the expression of ADAM12 by protein-level experiments, which is consistent with the above findings.

## 5. Conclusions

In conclusion, our study shows that miRNA30b has the capacity to prevent breast cancer from spreading and progressing *in vitro*. The expression of ADAM12 is assumed to be downregulated in order for this to happen. As a result, miRNA30b represents a possible new target for the treatment of breast cancer.

## Data Availability

The datasets generated during or analyzed during the current study are available from the corresponding author upon reasonable request.
